# Development of Discordant Hypermetabolic Prostate Cancer Lesions in the Course of [^177^Lu]PSMA Radioligand Therapy and Their Possible Influence on Patient Outcome

**DOI:** 10.3390/cancers13174270

**Published:** 2021-08-25

**Authors:** Philipp E. Hartrampf, Constantin Lapa, Sebastian E. Serfling, Andreas K. Buck, Anna Katharina Seitz, Philipp T. Meyer, Juri Ruf, Kerstin Michalski

**Affiliations:** 1 Department of Nuclear Medicine, University Hospital Wuerzburg, Oberdürrbacherstraße 6, 97080 Würzburg, Germany; Serfling_S1@ukw.de (S.E.S.); Buck_A@ukw.de (A.K.B.); 2 Nuclear Medicine, Medical Faculty, University of Augsburg, Stenglinstraße 2, 86156 Augsburg, Germany; Constantin.Lapa@uk-augsburg.de; 3 Department of Urology and Paediatric Urology, University Hospital Wuerzburg, Oberdürrbacherstraße 6, 97080 Würzburg, Germany; Seitz_A3@ukw.de; 4 Department of Nuclear Medicine, Medical Center, Faculty of Medicine, University of Freiburg, Hugstetter Straße 55, 79106 Freiburg, Germany; philipp.meyer@uniklinik-freiburg.de (P.T.M.); juri.ruf@uniklinik-freiburg.de (J.R.); kerstin.michalski@uniklinik-freiburg.de (K.M.)

**Keywords:** PSMA, FDG, PET/CT, prostate cancer, radioligand therapy

## Abstract

**Simple Summary:**

Discordant FDG-positive but PSMA-negative (FDG+/PSMA−) metastases constitute a negative prognostic marker of overall survival in patients undergoing PSMA radioligand therapy (RLT). The aim of this analysis was to investigate the prognostic implications of new FDG+/PSMA− lesions, which occur during or after PSMA RLT. In a retrospective bicentric analysis of 32 patients undergoing PSMA RLT and follow-up dual tracer staging with PSMA and FDG PET/CT, FDG+/PSMA− lesions occurred in a limited number of patients. However, the presence of FDG+/PSMA− lesions appears not to have a significant impact on the OS, but further studies are needed to establish the clinical relevance of such lesions.

**Abstract:**

Introduction: Positron emission tomography/computer tomography (PET/CT) targeting the prostate-specific membrane antigen (PSMA) is crucial for the assessment of adequate PSMA expression in patients with metastatic castration-resistant prostate cancer (mCRPC) prior to PSMA radioligand therapy (PSMA RLT). Moreover, initial dual tracer staging using combined PSMA and [^18^F]fluorodeoxyglucose (FDG) PET/CT provides relevant information, since discordant FDG-positive but PSMA-negative (FDG+/PSMA−) lesions constitute a negative prognostic marker of overall survival (OS) after PSMA RLT. However, little is known about the prognostic implications of dual tracer imaging for restaging at follow-up. The aim of this analysis was to investigate the prognostic implications of new FDG+/PSMA− lesions during or after PSMA RLT. Methods: This bicentric analysis included 32 patients with mCRPC who underwent both FDG and PSMA PET/CT imaging after two or four cycles of PSMA RLT. Patients with FDG+/PSMA− lesions prior to PSMA RLT were not considered. The presence of FDG+/PSMA− lesions was assessed with follow-up dual tracer imaging of patients after two or four cycles of PSMA RLT. Patients with at least one new FDG+/PSMA− lesion were compared to patients without any FDG+/PSMA− lesions at the respective time points. A log-rank analysis was used to assess the difference in OS between subgroups. Results: After two cycles of PSMA RLT, four of 32 patients (13%) had FDG+/PSMA− metastases. No significant difference in OS was observed (*p* = 0.807), as compared to patients without FDG+/PSMA− lesions. Follow-up dual tracer imaging after the 4th cycle of PSMA RLT was available in 18 patients. Of these, four patients presented with FDG+/PSMA− findings (*n* = 2 already after two cycles). After the fourth cycle of PSMA RLT, no significant difference in OS was observed between patients with and without FDG+/PSMA− lesions (*p* = 0.442). Conclusion: This study shows that FDG+/PSMA− lesions develop in a limited number of patients undergoing PSMA RLT. Further studies are needed to establish the clinical relevance of such lesions.

## 1. Introduction

In the last decade, several strategies of drug treatment have successfully prolonged survival in patients with metastatic castration-resistant prostate cancer (mCRPC), especially next-generation androgen receptor signaling inhibitors such as abiraterone and enzalutamide [1,2], or chemotherapy with docetaxel and cabazitaxel [3,4,5]. Furthermore, promising results for prostate-specific membrane antigen (PSMA-)-directed radioligand therapy (RLT) with 177Lu-labelled PSMA-ligands in advanced disease stages have been demonstrated [6,7,8]. The most important selection criterion for PSMA RLT is sufficient expression of PSMA in tumor manifestation sites, as assessed by PSMA positron emission tomography/computer tomography (PSMA PET/CT) [9]. However, patients scheduled for PSMA-directed RLT are often heterogeneous both in terms of prior treatment as well as tumor biology. Therefore, additional [^18^F]fluorodeoxyglucose (FDG) PET/CT staging becomes increasingly important, as it appears to be useful for the detection of more aggressive disease [10,11] and may help in predicting and optimizing response rates to RLT [12,13]. The LuPSMA Trial, which showed encouraging results for patients receiving RLT [14,15] excluded patients in the case of discordant FDG-positive but PSMA-negative (FDG+/PSMA−) lesions. These subjects showed a very poor overall survival (OS) under standard of care [16]. Regarding patients with FDG+/PSMA− lesions on initial dual tracer imaging, our group recently showed a reduced OS compared to patients without FDG+/PSMA− lesions undergoing RLT [17]. Furthermore, as tumor PSMA expression may decrease or be lost during several lines of treatment, additional FDG PET/CT scanning may play an important role in the detection of such lesions over the course of therapy [14,18].

Up to now, dual tracer imaging has been mainly used for risk stratification at baseline staging prior to PSMA RLT. The aim of this bi-centric retrospective study was to evaluate the prognostic implications of newly developed FDG+/PSMA− lesions on dual tracer follow-up imaging in patients undergoing PSMA-directed RLT. Therefore, FDG and PSMA PET/CT scans of patients after two or four cycles of PSMA-directed RLT were analyzed, and the results correlated to OS.

## 2. Materials and Methods

### 2.1. Patient Cohort

All patients who started their treatment with PSMA-directed RLT for mCRPC during the period between August 2018 and January 2020, and who underwent PSMA and FDG PET/CT before therapy initiation, were screened for eligibility at both participating sites (University Hospital Würzburg, Wuerzburg, Germany. University Hospital Freiburg, Freiburg, Germany). PSMA ligand PET/CT was performed to assess eligibility for PSMA-directed RLT. In the case of adequate PSMA expression, the patients routinely underwent a subsequent FDG PET/CT scan to assess the presence of PSMA-negative metastases. Patients were included only if staging with both tracers was available after the 2nd therapy cycle. An additional follow-up dual tracer staging after the 4th cycle was optional. Patients with known FDG+/PSMA− lesions already on initial dual tracer staging before PSMA-directed RLT were excluded from the current analysis to prevent the bias of the known prognostic impact of initial FDG+/PSMA− lesions. An analysis of the initial staging using PSMA and FDG PET/CT before PSMA-directed RLT has been previously published for most of the patients [17]. Further exclusion criteria were missing survival data, an intraindividual switch of PSMA ligands (i.e., from [^68^Ga]Ga-PSMA-11 to [^18^F]PSMA-1007) during the course of therapy, and a time interval of ≥ 3 months between PET/CT and application of the therapy. The median time of follow-up of surviving patients after two cycles of PSMA RLT was 15 (95% confidence interval (CI) 12.8–17.2) months. The study was approved by the local ethics committees (University Hospital of Freiburg: protocol No. 251/17; University Hospital of Wuerzburg: protocol No. 2019081501).

### 2.2. Imaging and Treatment Protocol

Imaging and treatment protocols have already been described elsewhere [17]. In brief, whole-body PET scans were acquired using a PET/CT scanner with either full-dose contrast-enhanced diagnostic CT (PSMA) or low-dose CT (FDG) for attenuation correction and anatomical co-registration. Both PET/CT studies were performed on two separate days. Standardized institutional protocols for RLT work-up were applied. In-house labeling was carried out for [^177^Lu]-labelled PSMA ligands [^177^Lu]Lu-PSMA I&T (Wuerzburg, Germany), and [^177^Lu]Lu-PSMA 617 (Freiburg, Germany). The standard PSMA RLT protocol consisted of infusion of 6.0 GBq of the radioligand every 6–8 weeks with up to a maximum of 4 cycles depending on response to treatment.

### 2.3. Image Analysis

PET/CT images were retrospectively analyzed using commercial software packages (Wuerzburg: Syngo.via; VB30A, Siemens Healthcare, Erlangen, Germany; Freiburg: IMPAX EE; Agfa Health Care, Bonn, Germany), as already described elsewhere [17]. All lesions with non-physiological higher uptake of the PSMA ligand or FDG than the physiological background were rated as PSMA- or FDG-positive, respectively. Images were visually evaluated independently on each participating site by two nuclear medicine physicians (PH and KM) using two categories: presence of one or more discordant lesions (FDG-positive but PSMA-negative) or absence of FDG+/PSMA− lesions. In addition, the location and the number of the FDG+/PSMA− lesions were noted. In case of visceral FDG+/PSMA− metastases, the visibility on the CT was noted. The extent of metastases on PSMA PET/CT was classified following the PROMISE Classification [19] with modifications: low tumor burden (≤3 metastases), intermediate tumor burden (>3 but <10 metastases), high tumor burden (≥10 metastases), and diffuse bone marrow involvement.

### 2.4. Statistical Analysis

Statistical analyses were performed using SPSS software version 27.0 (IBM, Armonk, NY, USA). Descriptive data are presented as mean ± standard deviation and range in parentheses. Survival data was analyzed by Kaplan–Meier curves and log-rank comparison. OS was calculated starting with the date of the PSMA PET/CT after PSMA RLT (i.e., after two or four cycles) and is presented as the median and 95% confidence interval (CI) in square brackets. A *p*-value less than 0.05 was considered statistically significant.

## 3. Results

### 3.1. Patients’ Characteristics

**Figure 1:**
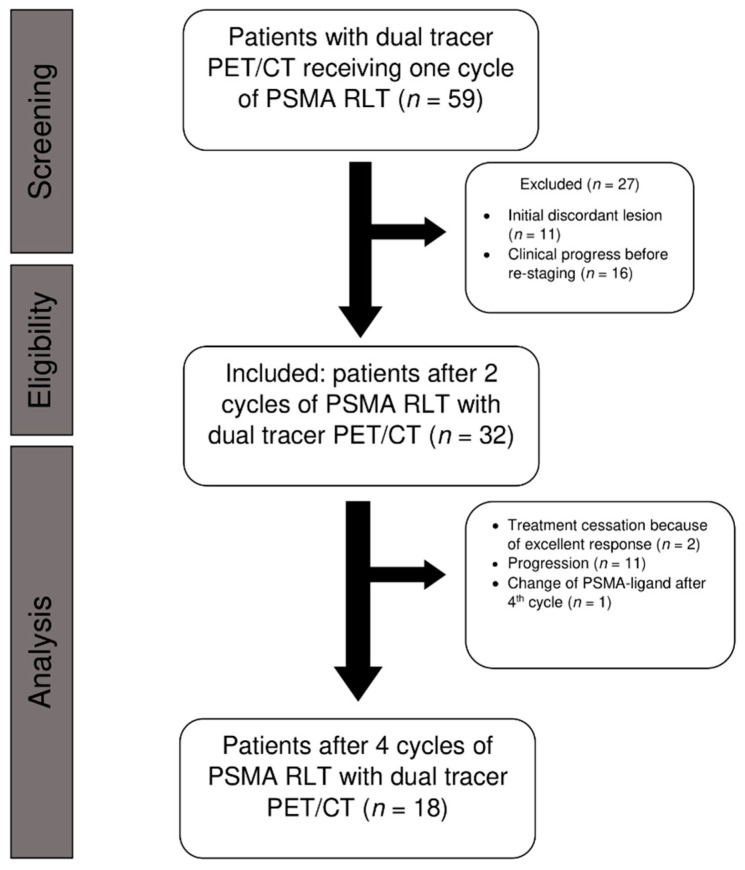
Flow diagram of patients included in this analysis undergoing PSMA radioligand therapy (RLT) and dual tracer PET/CT.

A total of 59 patients (Wuerzburg: *n* = 31; Freiburg: *n* = 28) who underwent dual tracer PET/CT prior to scheduled PSMA-directed RLT were screened for eligibility and 32 patients (Wuerzburg: *n* = 15; Freiburg: *n* = 17) were finally included into this analysis. For 18 of these patients (Wuerzburg: *n* = 10; Freiburg: *n* = 8) dual tracer staging was also available after the fourth treatment cycle. Figure 1 shows the inclusion process. Detailed characteristics of all included patients are given in Table 1.

On initial PSMA PET/CT, a low tumor burden was found in one patient (3%). In seven patients, an intermediate tumor burden was recorded (22%). The majority (*n* = 21; 66%) of patients showed a high tumor burden. Three patients presented with diffuse bone marrow involvement (9%). In total, 107 cycles of RLT were administered with a mean activity of 5.9 ± 0.6 (2.1–6.4) GBq per cycle.

### 3.2. Dual Tracer Staging after Two Cycles of PSMA RLT

After two cycles of PSMA-directed RLT, four of 32 patients (13%) showed FDG+/PSMA− metastases in the following organ systems: lymph nodes (*n* = 1), bones (*n* = 1), liver (*n* = 1), as well as liver and peritoneum (*n* = 1). The median number of FDG+/PSMA− findings was 2 (1–4). The liver metastases were only seen on FDG PET, not on the CT scan (one of these patients was examined with an unenhanced CT because of hyperthyroidism; Table 2).

**Figure 2:**
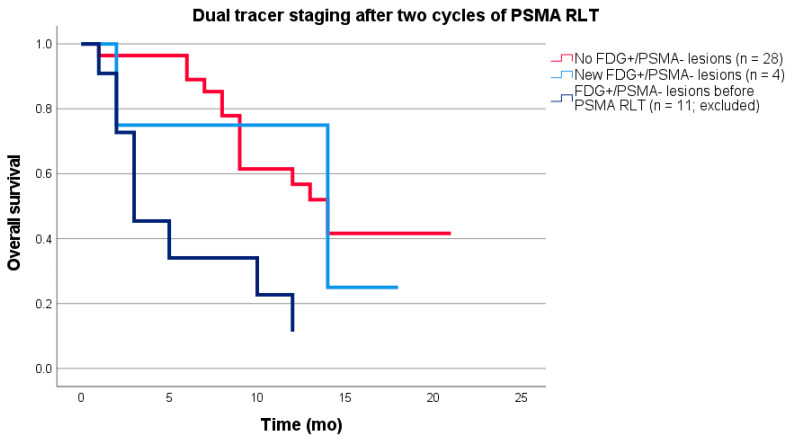
Kaplan–Meier curves of overall survival (OS). No significant difference for OS was found between the four patients with FDG+/PSMA− findings on dual tracer PET/CT after two cycles of PSMA radioligand therapy (RLT) (light blue; median OS 14 (95% CI 3.8–24.2) months) and the 28 patients without FDG+/PSMA− lesions (red; median OS 14 (95% CI 11.3–16.7) months; log rank test, *p* = 0.807). Patients with FDG+/PSMA− findings before PSMA RLT (dark blue, *n* = 11; excluded from the current analysis) showed a significantly shorter OS after two cycles than the 32 patients included in this study with a median OS of 3 (95% CI 0.7–5.3) months (log rank test, *p* < 0.001).

OS of all patients (*n* = 32) after two cycles of PSMA-directed RLT was 14 (95% CI 12.9–15.1) months. No significant difference for OS was found between the four patients with FDG+/PSMA− findings on restaging dual tracer PET/CT (median OS 14 (95% CI 3.8–24.2) months) and the 28 patients without FDG+/PSMA− lesions (median OS 14 (95% CI 11.3–16.7) months; *p* = 0.807; Figure 2).

### 3.3. Dual Tracer Staging after Four Cycles of PSMA RLT

**Figure 3:**
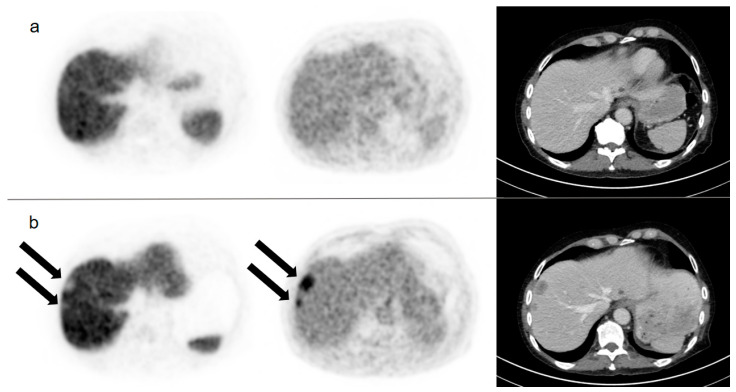
Corresponding axial slices of [^18^F]PSMA-1007 PET (first column), FDG PET (second column), and contrast enhanced CT (third column) of a 69-year old patient before PSMA radioligandtherapy (**a**) and after four cycles of PSMA radioligandtherapy with new FDG+/PSMA− liver metastases (black arrows); (**b**) fixed inverse gray-scale are displayed with SUV window setting from 0 to 20 ([^18^F]PSMA-1007 PET) and 0 to 5 (FDG PET), respectively.

Four of 18 patients (22%) showed FDG+/PSMA− findings on dual tracer staging after four cycles of PSMA RLT. Of these, two patients had already presented with FDG+/PSMA− metastases on dual tracer staging after the 2nd cycle and showed progression of these lesions after the 4th cycle (of note, other PSMA+ lesions also progressed). Two patients presented with FDG+/PSMA− findings for the first time. Figure 3 shows a patient with new FDG-/PSMA+ liver metastases after four cycles of PSMA RLT.

**Figure 4:**
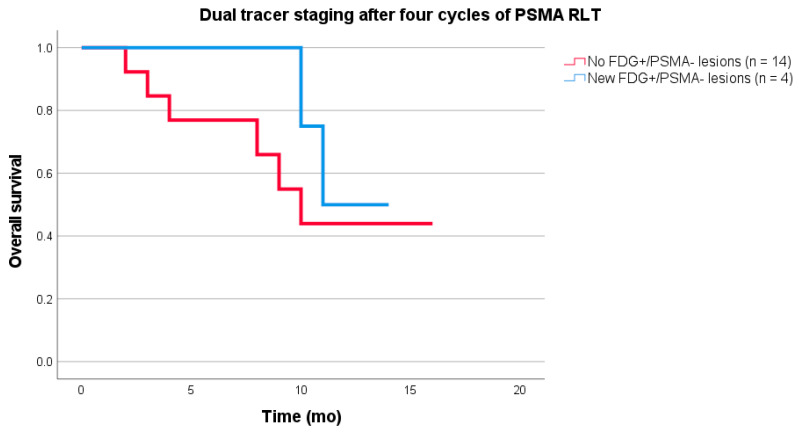
Kaplan–Meier curves of overall survival (OS). No significant difference for OS was found between the four patients with FDG+/PSMA− findings on dual tracer PET/CT (median OS not reached at the end of the follow-up period) after four cycles of PSMA radioligand therapy (RLT) and the 14 patients without FDG+/PSMA− lesions (median OS 10 (95% CI 7.2–12.8) months; *p* = 0.442).

The following organ systems were affected: bones (*n* = 1), lymph nodes (*n* = 1), liver (*n* = 1), as well as liver and peritoneum (*n* = 1). The median number of FDG+/PSMA− findings was five (1–9). In one patient, the hepatic lesions were only found on the FDG PET scan, but not on the unenhanced CT scan. The peritoneal carcinomatosis in the same patient, as well as the liver metastases in the other patient, were visible on both FDG PET and CT (Table 2). The OS of all patients (*n* = 18) calculated after four cycles of PSMA-directed RLT was 11 (95% CI 8.8–13.2) months. No significant difference for OS was found between the four patients with FDG+/PSMA− findings on dual tracer PET/CT after the fourth cycle (median OS not reached at the end of the follow-up period) and the 14 patients without FDG+/PSMA− lesions (median OS 10 (95% CI 7.2–12.8) months; *p* = 0.442, Figure 4).

## 4. Discussion

While the prognostic relevance of FDG+/PSMA− PET-findings prior to PSMA RLT has recently been demonstrated [17], the present analysis focused on the development FDG+/PSMA− findings in the course of PSMA-directed RLT and assessed the potential prognostic value of repeated dual tracer imaging.

Rosar et al. studied 52 patients with dual tracer PET/CT over the course of PSMA-directed RLT, but detailed information about the time points or initial imaging was not given. FDG+/PSMA− findings were found in 28 of 52 patients (54%) [20], which is clearly higher than in our study cohort with 4 of 32 patients (13%) after the second cycle or 4 of 18 patients (22%) after the fourth cycle of PSMA RLT. This could be due to the fact that we excluded patients with FDG+/PSMA− lesions on initial dual tracer staging before PSMA RLT in our analysis. The appearance of FDG+/PSMA− lesions under PSMA RLT might reflect genomic dedifferentiation as a possible resistance mechanism to PSMA-directed therapy. Surprisingly, the presence of FDG+/PSMA− findings after the second and fourth cycle of PSMA RLT turned out not to be a prognostic marker in our study cohort. This might suggest that the assessment of an additional FDG PET/CT scan may be only reasonable for initial staging before PSMA RLT [17]. However, these results are limited due to the small number of patients in our patient cohort and need further investigations. Results of Emmett et al. [21] indicate that other parameters (such as the maximum standardized uptake value) and volume or site of disease on FDG PET are not prognostic markers for serological response, neither on initial staging nor after completion of therapy.

However, in terms of diagnostic accuracy, FDG PET might help to detect liver metastases, especially in [^18^F]PSMA-1007 PET scans, which can be false negative due to the high physiological hepatic tracer uptake [22]. Although previous analyses have shown that the presence of visceral metastases, especially liver metastases, results in poor survival for patients undergoing RLT [17,23,24], disease control of patients with PSMA-positive liver metastases is still possible in approximately half of cases (46%) [25]. Therefore, the detection of PSMA-negative liver metastases is very important regarding possible discontinuation of PSMA-directed RLT. We observed one patient with liver metastases found only by FDG PET on follow-up imaging, but not with the contrast-enhanced CT scan. However, this patient was examined with [^18^F]PSMA-1007 PET/CT, and the FDG+/PSMA− assessment might be due to the hepatobiliary excretion of [^18^F]PSMA-1007 [22]. Thus, if the possible detection of PSMA-negative liver metastases justifies the routine use of FDG PET/CT remains questionable.

Apart from that, in some cases, it might be useful to perform additional FDG PET/CT, if neuroendocrine dedifferentiation is suspected (e.g., loss of tracer-uptake on PSMA PET/CT, increase of neuron-specific enolase [20], PSA negative follow-up) for choosing the right therapy management, but we cannot provide data on this issue. FDG PET/CT could then also be used for therapy monitoring. Apart from FDG PET, nomograms of PSMA PET scans might be another possible tool for an improved patient selection for staging and restaging of patients with (metastasized) prostate cancer [26,27].

Patients showed a median survival of 14 months after the second cycle and 11 months after the fourth cycle. This is apparently longer when compared to the OS of 11 months for all patients treated with RLT after beginning of the first cycle, which was published recently [17]. This is probably due to a selection bias of our study, which analyzes only those patients without initial FDG+/PSMA− lesions and known reduced response to therapy. Furthermore, our analysis does not include the data of 16 patients who were treated with one or two cycles of PSMA RLT, but who did not undergo PET/CT restaging due to clinical disease progression with discontinuation of PSMA RLT. Therefore, the percentage of patients with FDG+/PSMA− metastases can be underestimated.

Additional limitations of this retrospective study include the small sample size, especially for follow-up dual PET/CT after the fourth cycle of PSMA RLT, which included only 18 patients. However, this study constitutes the first systematic analysis of FDG+/PSMA− lesions in sequential dual tracer PET/CT in patients undergoing PSMA RLT. No histopathological proof of FDG−/PSMA+ metastases was undertaken and a false positive rating (i.e., in case of an inflammation) cannot be completely ruled out, although it seems unlikely as all scans were also evaluated with regard to their CT scan. PET/CT examinations using both [^68^Ga]- and [^18^F]-labelled PSMA ligands were included, as both hospitals changed the radiolabeling procedure during the study period. Despite this fact, the included follow-up PSMA PET scans of a given patient were strictly performed with the same PSMA ligand only. Moreover, in order to deal with potential differences of the PSMA ligands, a visual categorization system as described before was used [17]. Within this framework, the impact of different PET/CT scanners used at the two institutions for the visual assessment is probably negligible.

## 5. Conclusions

This study shows that FDG+/PSMA− lesions develop in a limited number of patients undergoing PSMA RLT. Further studies are needed to establish the clinical relevance of such lesions.

## Figures and Tables

**Table 1:** Patients’ characteristics.

	After 2 Cycles of PSMA RLT (*n* = 32)		After 4 Cycles of PSMA RLT (*n* = 18)	
	Mean ± SD (Range)		Mean ± SD (Range)	
Age (years)	72.6 ± 8.9 (46–90)		75.3 ± 9.8 (46–85)	
Time since diagnosis of prostate cancer (years)	8.1 ± 6.6 (2–26)		10.1 ± 7.8 (2–26)	
Gleason score at diagnosis	(7–10) *		(7–9) **	
ECOG before PSMA RLT	(0–2)		(0–2)	
PSA before PSMA RLT [ng/mL]	389 ± 709 (5–2650)		384 ± 670 (5–2650)	
Sites of disease before PSMA RLT	n	%	n	%
Prostate/local	13	41	7	39
Lymph node	17	53	9	50
Bone	30	94	18	100
Liver	3	9	1	6
Lung	4	13	3	17
Other	3	9	2	11
Previous treatment	n	%	n	%
Prostatectomy	17	53	11	61
Radiotherapy to prostate/prostate bed	15	47	9	50
ADT	32	100	18	100
Abiraterone	25	78	12	67
Enzalutamide	19	59	13	72
Docetaxel	21	66	11	61
Cabazitaxel	7	22	4	22
Other	6	19	3	11

ECOG: performance status according to Eastern Cooperative Oncology Group; PSA: prostate specific antigen, ADT: androgen deprivation therapy, PSMA RLT: prostate-specific membrane antigen-targeted radioligand therapy; other therapies: selective internal radiation therapy, PSMA RLT, Carboplation, Estramustin, Prostvac, Gemcitabine, Olaparib; other sites of disease: adrenal gland, testis, peritoneal carcinomatosis; * unknown in five patients; ** unknown in four patients.

**Table 2:** Findings of the patients with discordant lesions after two and four cycles of PSMA RLT.

Pat. #	Before PSMA RLT	After 2 Cycles of PSMA RLT			After 4 Cycles of PSMA RLT		
	# of Metastases on PSMA PET/CT	# of Metastases on PSMA PET/CT	# of Discordant Lesions	Discordant Organs	# of Metastases on PSMA PET/CT	# of Discordant Lesions	Discordant Organs
3	>10	DBMI	1	OSS	−	−	−
4	DBMI	DBMI	2	HEP *	−	−	−
7	>10	>10	n.a.	n.a.	>10	1	OSS
10	>3 but <10	>3 but <10	4	HEP **, PeC	>10	7	HEP **, PeC
14	>10	>10	n.a.	n.a.	>10	3	HEP
23	>10	>10	1	LN	>10	9	LN

Explanations: PSMA = prostate specific membrane antigen; RLT = radioligand therapy; DBMI = diffuse bone marrow involvement; OSS = bone; LN = lymph node; HEP = liver; PeC = peritoneal carcinomatosis; n.a. = not applicable; * only visible on FDG PET, not on the contrast-enhanced CT scan, ** only visible on FDG PET, not on the unenhanced CT scan.

## Data Availability

The main data presented in this study are available in the article. Detailed information about the image analysis or the overall survivals of the subjects presented in this study are available on request from the corresponding author.
